# National Infant Immunization Week — April 20–27, 2013

**Published:** 2013-04-19

National Infant Immunization Week (NIIW) will be observed April 20–27, 2013. An annual event since 1994, NIIW brings together local and state health departments, national immunization partners, and health-care professionals across the country to hold community activities and events highlighting the importance of protecting infants from vaccine-preventable diseases through immunization.

Although immunization rates for vaccines routinely recommended for children remain at or near record highs, recent outbreaks of measles and pertussis in the United States underscore the importance of maintaining high immunization rates by successfully addressing parents’ questions and concerns about childhood vaccines (1). This year, CDC developed educational and promotional materials to remind parents of the importance of vaccinating their children according to the recommended immunization schedule. For the second consecutive year, NIIW will be observed simultaneously with World Immunization Week, an initiative of the World Health Organization to promote immunization and advance equity in the use of vaccines and universal access to vaccination services. Additionally, the CDC Foundation and CDC will recognize recipients of the second annual CDC Childhood Immunization Champion Award, which recognizes individuals for their contributions to public health through their work in childhood immunizations.

Additional information about NIIW is available at http://www.cdc.gov/vaccines/events/niiw. Additional information about World Immunization Week is available at http://www.who.int/campaigns/immunization-week/2013/en/index.html.
